# Predictors of Behavioral and Psychological Symptoms in Community-Dwelling Older Adults With Dementia

**DOI:** 10.1093/geroni/igab046.2449

**Published:** 2021-12-17

**Authors:** Bada Kang, Eunhee Cho, Sujin Kim, Sinwoo Hwang, Eunji Kwon, Seok-Jae Heo, Jun Hong Lee, Byoung Seok Ye

**Affiliations:** 1 Mo-Im Kim Nursing Research Institute, Yonsei University College of Nursing, Seoul, Seoul-t'ukpyolsi, Republic of Korea; 2 Yonsei University College of Nursing, Seoul, Seoul-t'ukpyolsi, Republic of Korea; 3 Yonsei University, Seoul, Seoul-t'ukpyolsi, Republic of Korea; 4 Korea Armed Forces Nursing Academy, Daejeon, Taejon-jikhalsi, Republic of Korea; 5 Yonsei University Graduate School, Seoul, Seoul-t'ukpyolsi, Republic of Korea; 6 National health insurance service Ilsan hospital, Goyang, Kyonggi-do, Republic of Korea

## Abstract

Although disclosing the predictors of different behavioral and psychological symptoms of dementia (BPSD) is the first step in developing person-centered interventions, the current understanding is limited as it considers BPSD as a homogenous construct, not accounting for its heterogeneity. Therefore, this study explored the predictors of BPSD subsyndromes, and built prediction models for these subsyndromes in community-dwelling older adults with dementia in Korea. This prospective study consisted of a two-wave dataset. We fit the generalized linear mixed models using Wave 1 data (N = 145) and then validated them using Wave 2 data (N = 59). BPSD and their proximal factors were assessed on a daily basis using diaries written by family caregivers. Sleep and activity levels were objectively measured using actigraphy. The amount of nighttime sleep hours was significantly associated with next-day sleep and nighttime behaviors (OR = 0.87; p = 0.005), with the amounts of energy expenditure showing significant association with euphoria/elation (OR = 0.02; p = 0.019). All subsyndromes except euphoria/elation were found to be significantly associated with either hunger, thirst, urination, or bowl movement; with all BPSD showing a significant association with environmental changes. We also found several background factors, including premorbid personality and taking sedatives as predictors for specific subsyndromes. The area under the receiver operating characteristic curve scores for the data were greater than 0.9 and 0.8 in Waves 1 and 2, respectively, across all subsyndromes. Prediction models for BPSD will help in the development of symptom-targeted, individualized interventions.

